# How Packaging of Information in Conversation Is Impacted by Communication Medium and Restrictions

**DOI:** 10.3389/fpsyg.2021.594255

**Published:** 2021-04-16

**Authors:** Sarah A. Bibyk, Leslie M. Blaha, Christopher W. Myers

**Affiliations:** 711th Human Performance Wing, Airman Systems Directorate, Air Force Research Laboratory, Wright-Patterson Air Force Base, Dayton, OH, United States

**Keywords:** conversation, synthetic teammate, information packaging, speech, text, chat

## Abstract

In team-based tasks, successful communication and mutual understanding are essential to facilitate team coordination and performance. It is well-established that an important component of human conversation (whether in speech, text, or any medium) is the maintenance of common ground. Maintaining common ground has a number of associated processes in which conversational participants engage. Many of these processes are lacking in current synthetic teammates, and it is unknown to what extent this lack of capabilities affects their ability to contribute during team-based tasks. We focused our research on how teams *package* information within a conversation, by which we mean specifically (1) whether information is explicitly mentioned or implied, and (2) how multiple pieces of information are ordered both within single communications and across multiple communications. We re-analyzed data collected from a simulated remotely-piloted aerial system (RPAS) task in which team members had to specify speed, altitude, and radius restrictions. The data came from three experiments: the “speech” experiment, the “text” experiment, and the “evaluation” experiment (which had a condition that included a synthetic teammate). We asked first whether teams settled on a specific routine for communicating the speed, altitude, and radius restrictions, and whether this process was different if the teams communicated in speech compared to text. We then asked how receiving special communication instructions in the evaluation experiment impacted the way the human teammates package information. We found that teams communicating in either speech or text tended to use a particular order for mentioning the speed, altitude, and radius. Different teams also chose different orders from one another. The teams in the evaluation experiment, however, showed unnaturally little variability in their information ordering and were also more likely to explicitly mention all restrictions even when they did not apply. Teams in the speech and text experiments were more likely to leave unnecessary restrictions unmentioned, and were also more likely to convey the restrictions across multiple communications. The option to converge on different packaging routines may have contributed to improved performance in the text experiment compared some of the conditions in the evaluation experiment.

## 1. Introduction

As the capabilities of Artificial Intelligence (AI) increase and diversify, its application to teams will become more and more ubiquitous. Significant advancements in AI and a deeper understanding of human cognition remain for machines to move beyond supportive technology and become “teammates.” Such *synthetic teammates* must not only be able to comprehend and carry out instructions from human team members, but they must also be capable of coordinating with them (Gorman et al., 2010; Cooke et al., 2013; Mancuso et al., 2014; McNeese et al., 2018; Grimm et al., 2019). Human teams coordinate through communication; however, fundamental unanswered questions persist as to how precisely conversation coordinates both knowledge and actions, and what conversational behaviors specifically contribute most to team performance and should be built into synthetic teammates.

It is well-established that human conversation is not equivalent to two interlocutors producing and interpreting utterances in isolation, as if they were recording a monologue or listening to that monologue separate from each other. Conversational participants engage in a number of behaviors and processes to ensure they are correctly interpreting the utterances, and to correct any misunderstandings or misinterpretations that arise. In other words, participants in a conversation must work to maintain *common ground* (e.g., Stalnaker, 1978; Clark and Wilkes-Gibbs, 1986), the set of beliefs, knowledge, and representations that allow participants to properly situate and interpret utterances with respect to the current conversation. For example, consider a single text message sent by a Navigator in a remotely-piloted aerial system synthetic task environment (RPAS-STE) through a chat window interface:

Navigator: s 200-500 a 1000-2000 r 5

Without proper context, the meaning of such a message might be rather opaque. However, the Navigator's teammates who share common ground know to interpret this message as meaning:

Navigator: For the next waypoint, speed restrictions for the aircraft are between 200 and 500 knots, altitude restrictions are between 1000 and 2000 feet, and the effective radius for entry is 5 nautical miles.

In order to improve synthetic teammates' abilities to both interpret language and maintain common ground, current research has explored a number of options, including developing situated dialog systems that can interpret not only an utterance's meaning but also its function within the discourse (e.g., Bonial et al., 2019, 2020), as well as systems that include shared mental models of teammates' abilities and current task states (e.g., Gervits et al., 2020) and systems for managing dialogue based on agents' situation representation (Rodgers et al., 2013). Such increasingly sophisticated systems for representing both language and the task environment will likely be key to improving teammates' abilities to communicate and maintain common ground. One prominent theory of dialogue, however, argues that alignment at high-level mental representations is facilitated by alignment within the linguistic content itself (Pickering and Garrod, 2004). This alignment often takes the form of repetition of linguistic material both within and across speakers, also called *entrainment*, and has been demonstrated empirically for a variety of linguistic structures. Entrainment has been found for particular words or labels (e.g., “speed” in lieu of “airspeed”), also called *conceptual pacts* (Brennan and Clark, 1996), and it has also been found for larger chunks of language such as syntactic structures and phrases (Branigan et al., 2000, 2003). Newer research suggests, however, that linguistic alignment in general may not be the best predictor of team performance (e.g., Rothwell et al., 2017). When and how entrainment happens is likely complex; for example there is some evidence that lexical entraiment is affected by the type of dialogue act (Mizukami et al., 2016). If we are to include entrainment as a feature in synthetic teammates, we must better understand what it is beneficial for them to entrain on and when.

When coordinating on a task through language, interlocutors must make choices not only about their lexical items and syntactic structures, but also make decisions about how to *package* information more generally, including how to *order* information and whether to explicitly *mention* information. Information packaging (also called information structure) refers to how linguistic material is organized both within and across utterances. It often contributes to differences in higher level meanings (rather than “literal” or semantic meaning), such as conveying information that is *new* in a sentence, in contrast to information that is *given* (see Krifka, 2008, for an overview). For example, our Navigator may choose from a number of possible orders, such as:

Alternative 1: a 1000-2000 r 5 s no restrictions
Alternative 2: a 1000-2000 s no restrictions r 5

which can roughly all be interpreted in the same way:

Navigator: For the next waypoint, there are no speed restrictions for the aircraft, altitude restrictions are between 1000 and 2000 feet, and the effective radius for entry is 5 nautical miles.

though the information has been structured differently.

Our Navigator may also choose to explicitly mention that certain restrictions do not apply, or leave them unmentioned and rely on the other teammates to infer that they do no apply:

Alternative 1: a 1000-2000 r 5 s none
Alternative 2: a 1000-2000 r 5

Interlocutors commonly shorten or drop linguistic material once they have established common ground. For example, in the now well-established Tangram task (Clark and Wilkes-Gibbs, 1986), two participants must work together to develop names for images on a set of cards. One participant serves as the *Director*, and the other the *Matcher*; the Director's job is to instruct the Matcher in what order to arrange the cards. Neither participant can see each other, nor each other's cards. The images on the cards are composed out of Tangram shapes, and are difficult to describe. To solve the task, Directors typically begin by providing long descriptions in early trials (e.g., “the next one looks like a person who's ice skating, except they're sticking two arms out in front”; Clark and Wilkes-Gibbs, 1986), in order to *ground* the card to which they are referring (Clark and Schaefer, 1987, 1989; Clark and Brennan, 1991). Across repeated trials, however, they eventually shorten these descriptions to efficient labels (e.g., “the ice skater”). Conversational participants are thus able to become more efficient in their language use yet maintain understanding, but it is only through their mutual participation in these processes. Matchers who overhear an entire conversation, but are not able to actively participate themselves, do not perform as well at arranging the Tangram images (Schober and Clark, 1989).

It remains unknown whether information packaging will follow similar patterns. We sought to investigate whether teammates would converge on a specific routine for the packaging of information for the purposes of helping maintain common ground, similar to lexical and syntactic entrainment. Specifically, we asked (1) would they use a particular ordering of the speed, altitude and radius restrictions, and (2) would they continue to use this order even with waypoints that did not have all of the restrictions, or would they leave unused restrictions unmentioned.

We also sought to investigate how packaging of information might be affected by the communication medium. The processes for maintaining common ground and alignment that take place in speech communications are also present in text communications (e.g., Potts, 2012; Mills, 2014), however the difference in communication medium also leads to differences in grounding behaviors (Clark and Brennan, 1991). For many tasks, agents who communicate in speech are desirable, but are also challenging to implement (e.g., Cantrell et al., 2010; Veale and Scheutz, 2012; Scheutz et al., 2013), partially due to the limitations many Automatic Speech Recognition (ASR) systems still face (e.g., Georgila et al., 2020). An alternative is to have synthetic teammates and intelligent agents who communicate through text (e.g., Myers et al., 2019). Understanding how differences in the routinization of packaging play out in communicating in text compared to speech is important both for making improvements in how current text-based agents communicate and also for making future changes in the processes of these agents when they transition from text to speech communication.

Finally, given that it is in unknown if and how packaging of information is routinized during collaborative tasks, and it is unknown to what extent these processes may differ between speech and text, it is also unknown how the possibility of interacting with a text-based synthetic teammate may further alter these processes. We had access to a synthetic teammate that was capable of interpreting multiple packaging options, though it did not have completely unfettered natural language comprehension, and it had no entrainment capabilities. The ability to interpret multiple packaging options might suffice for facilitating entrainment in packaging for the human teammates, thus leading to no differences between a text condition without a synthetic teammate and a text condition with one. On the other hand, the restrictions that the teammate placed on communications might cause differences in entrainment between the teammates who communicated with the synthetic teammate and those who did not. Previous research suggests that participants may show stronger entrainment in syntax when communicating with a synthetic agent as compared to another human (e.g., Branigan et al., 2003).

To summarize, we seek to address whether packaging of information, specifically the linear ordering and explicit mention of information within communications, might become routinized while accomplishing a joint task in ways similar to how other aspects of language (e.g., lexical items and syntactic structures) are routinized in the service of maintaining common ground between interlocutors. We were interested in whether teammates might use both repetition and shortening/dropping of linguistic material. We also asked how communicating in text rather than speech might alter these processes, and further how the possibility of interacting with synthetic teammate might impact them. In the following section, we provide details on the empirical task, a set of three studies that communication data were collected from, and how the communication data were coded.

## 2. Materials and Methods

Data were collected from three different experiments using the same RPAS-STE. In the following subsections we provide details about the synthetic task environment for collecting the data, the procedure for each experiment, the number of participants from each experiment, and how the data were coded to conduct the analyses.

### 2.1. Remotely-Piloted Aerial System Synthetic Task Environment

To provide context on the communications discussed in the following analyses, the remotely-piloted aerial system synthetic task environment (RPAS-STE) will be discussed briefly. The RPAS-STE provides a testbed for the study of team cognition within a three-person team completing individual tasks toward a common goal (Cooke and Shope, 2004). In this STE, three participants coordinate to “fly” a simulated RPAS to photograph reconnaissance targets. Participants are assigned to the role of Pilot (Air Vehicle Operator; AVO), Photographer (Payload Operator; PLO), or Navigator (Data Exploitation Mission Planning and Communications Operator; Navigator/DEMPC). Participants are first trained on their specific tasks and then operate as a team to complete multiple 40-min reconnaissance missions to photograph stationary ground targets.

The RPAS-STE requires teammates to communicate ground target information to successfully achieve the team objective of taking as many reconnaissance photographs as possible in the allotted time. Critical ground target information that needed to be communicated from the Navigator/DEMPC and Photographer/PLO to the Pilot/AVO was the airspeed and altitude required to get a good photograph as well as the radius in nautical miles from the target that a photograph could be taken (i.e., the effective radius). If the altitude or airspeed were not within the required range for the reconnaissance target, or if the RPAS was outside of the effective radius, then the PLO/Photographer could not take a good photograph and the team would be docked points, lowering their team performance. Further, if the airspeed and altitude were in the process of changing to meet the communicated restrictions, then a photograph could not be taken. Consequently, the airspeed and altitude must be communicated with enough time to achieve RPAS performance prior to reaching the effective radius.

The RPAS-STE affords an opportunity to study both the ordering of information as well as whether information was explicitly mentioned or left implied. Teammates must decide (1) whether to combine all of the restrictions together into a single communication, (2) whether to maintain a consistent restriction order across multiple target waypoints, and (3) whether to mention restrictions that did not apply to a particular waypoint.

Each participant was seated in front of two computer monitors that displayed unique role information and common information about the vehicle's current attitude (heading, speed, altitude). Team member interaction could occur through either speech-based communications via a push-to-talk system or through text-based communications similar to instant messaging. Team and individual metrics have been designed, embedded in the RPAS-STE, and validated across 10 different experiments, leading to the development of a theory of interactive team cognition (Cooke et al., 2013). To objectively determine team performance, a composite outcome score is computed for teams at the end of each 40-min mission based on number of targets successfully photographed and the duration of different warnings and alarms incurred from each of the team members.

### 2.2. Experiments

Communication data from three different experiments all using the RPAS-STE were used in our analyses. In one experiment, participants communicated over a voice-based communication system (i.e., “speech experiment”; Gorman et al., 2010). In a second experiment, participants communicated over a text-based communication system (i.e., “text experiment”; Duran, 2010), and in a third experiment participants communicated over the same text-based communication system with three between-team conditions: (1) a group of naïve participants (“control condition”), (2) a group of naïve participants paired with a human expert in piloting the RPAS within the STE (“expert condition”), and (3) a group of naïve participants who worked with a computational cognitive model acting as a synthetic teammate and piloting the RPAS (“synthetic teammate condition”) (i.e., “evaluation experiment”; Myers et al., 2019). Experimental sessions for each of the three experiments took approximately 6.5 hours. Participants were randomly assigned to either the Pilot/AVO, Photographer/PLO, or Navigator/DEMPC role on the team with the exception of the Pilot/AVO position in the expert pilot and synthetic teammate conditions in the evaluation experiment.

### 2.3. Participants

In the speech experiment, 32 teams participated, with three participants in each team. In the text experiment 10 teams participated, three participants per team. In the evaluation experiment, 30 teams participated: 10 teams in the control condition, three participants per team; 10 teams in the expert pilot condition, two participants per team (the third member was the expert pilot who was the same for each team), and 10 teams in the synthetic teammate condition, two participants per team (the third member was the synthetic teammate pilot who was the same for each team). Each participant was compensated $10.00 per hour of participation. As an incentive, a $100.00 bonus was awarded to each participant of the highest performing team in their respective experiment.

### 2.4. General Procedure

Once team members were seated at their workstations, they signed a consent form, were given a brief overview of the study, and started training on their respective tasks: piloting, navigating, or photographing. During training, team members studied three PowerPoint training modules at their own pace and were tested with a set of multiple-choice questions at the end of each module. If responses were incorrect, experimenters provided assistance and explanations as to why their answers were incorrect and the reasoning behind the correct answers. Participants in the text versions of the experiment received training on the operation of the chat system and participants in the speech version received training on the operation of the push-to-talk system.

After training, teams started their first 40-min mission. All missions required the team to take reconnaissance photos of targets. However, the number of targets varied from mission to mission. Missions were completed either at the end of a 40-min interval or when team members believed that the mission goals had been completed. Immediately after each mission, participants were given the opportunity to view their performance scores. Participants viewed their team score, their individual score, and the individual scores of their teammates. The performance scores were displayed on each participant's computer and shown in comparison to the mean scores achieved by all other teams (or roles) who had participated in the experiment up to that point.

There were three critical differences across the experiments. First, the communication mode was either speech or text. Second, the inclusion of a synthetic teammate occurred as a condition in the evaluation experiment. Due to limitations of the synthetic teammate's ability to disambiguate references across multiple text communications, participants in all three conditions were instructed to communicate all waypoint information in a single text message (the waypoint name, waypoint type [target, entry, or exit], altitude restriction [if one was required], airspeed restriction [if one was required], and effective radius).

The final difference between the three experiments was the number of 40-min missions completed. In the speech experiment, participants completed eight normal load missions and ended with a high workload mission, totaling nine missions. The text and evaluation experiments both completed four normal missions and a fifth high workload mission. Only data from the first four missions from each experiment were considered for analyses. Table 1 summarizes the design differences across the three experiments.

**Table 1:** Differences between the speech, text, and evaluation experiments.

**Experiment**	**Conditions**	**Missions completed**	**Given instructions on packaging?**
Speech	N/A	9	No
Text	N/A	5	No
Evaluation	Naive pilot	5	Yes
	Expert pilot	5	Yes
	Synthetic pilot	5	Yes

### 2.5. Data Coding and Analysis

From the evaluation experiment, all transcripts from all teams for the first four missions were available for use (one team was excluded because they sometimes used a second language aside from English to communicate). From the text experiment, two missions (one from team 10 and one from team 20) had to be excluded due to issues in the output of the timestamps that rendered it difficult to reconstruct the original order of the messages. In the speech experiment, transcripts of the first four missions were not available for all teams (see Table 2 for a list of the transcripts that were available for analysis).

**Table 2:** Transcribed speech communication data available and analyzed from teams and missions.

**Team**	**Missions available**	**Missions analyzed**
12	1–4	1–4
14	1–9	1–4
17	3–4	3–4
18	4	4
19	1–5	1–4
20	1–9	1–4
21	1–5	1–4
22	1–4	1–4
23	1	1
24	1,3,4	1,3,4
25	1–5	1–4
26	1–4	1–4
27	1,2	1,2
30	1–4	1–4
31	1–9	1–4
32	1–4	1–4

**Figure 1:**
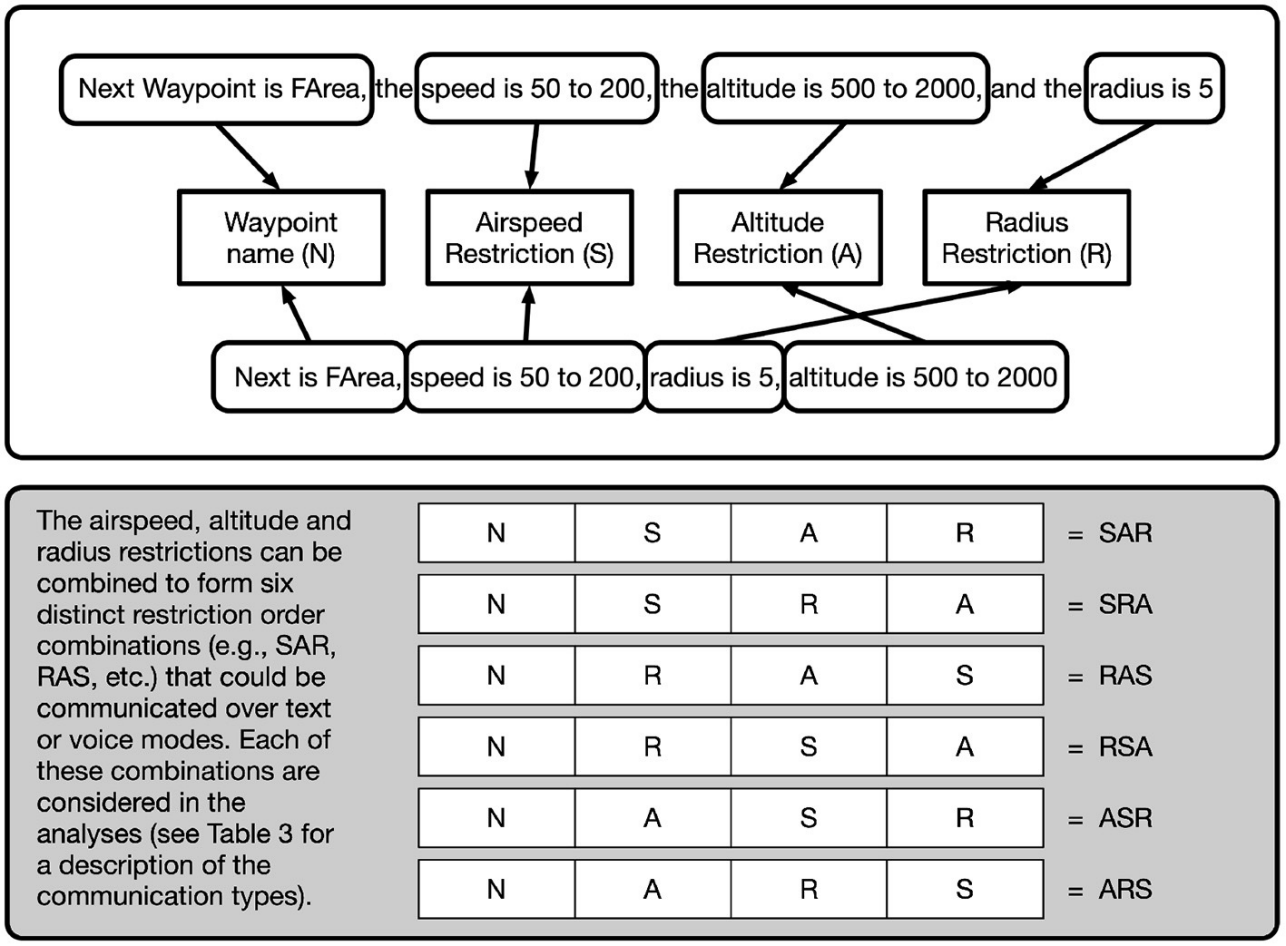
Deconstructing and identifying communications as an ordered set of waypoint restrictions: airspeed, altitude, and radius. In the top white box, example communications (text or speech) where aspects of a communication are tagged as being either a waypoint name, airspeed restriction, altitude restriction, or radius. The communications are deconstructed and tagged based on their restriction order (e.g., RSA, ARS, etc.; bottom gray box) for further analysis.

Because of the asymmetric nature of the roles of the teammates, only the Navigator/DEMPCs were particularly likely to mention all three kinds of restrictions (speed, altitude, and effective radius) at the same time for any one waypoint (see Figure 1). In future work, we would like to consider the complementary contributions from each of the teammates toward the grounding of the waypoints and their restrictions. Recall that our research questions included two aspects of packaging: (1) would the Navigator/DEMPCs entrain on a specific order of information (both within and across communications), and (2) would Navigator/DEMPCs explicitly mention restrictions that did not apply for a particular waypoint (for the purposes of maintaining the packaging routine) or would they drop the unused restrictions in order to shorten the communications over time. These two aspects determined the structure of the coding scheme described below.

#### 2.5.1. Full Canonical Communications

Our first data point of interest is what we will call a “single,” “full” communication. This is a communication sent by the Navigator/DEMPC (either in a single push-to-talk instance or as a single text message) that mentions explicitly (1) a particular waypoint by name, and (2) all three of the possible restrictions associated with that waypoint (i.e., altitude, airspeed, and effective radius required for obtaining a good photograph). For example:

Next waypoint is FArea, the speed is 50 to 200, the altitude 500 to 2000, and the radius is 5

This example provides an ideal test case for investigating whether Navigator/DEMPCs will entrain on a particular order for the restrictions. This type of communication was interpretable for the synthetic teammate, and was used as an example in training the human teammates for how to communicate with it. Hand correction of the data was done in cases where the Navigator/DEMPCs mispelled words or used abbreviations (e.g., “rad” or “r” for radius) or when they referred to a restriction only by its numeric value without naming which restriction it was explicitly. For example,

For next waypoint FArea, restrictions are 500 to 200, 500 to 1000, and 5

In some cases Navigator/DEMPCs did not explicitly mention within the communication which waypoint they were discussing, but if it was clear from the surrounding dialogue that they were talking about a single waypoint (and also clear which waypoint it was), such communications were included in the analyses.

#### 2.5.2. Communications With “Restrictions”

Because of the structure of the task, not all waypoints had restrictions on speed, altitude, and effective radius. In particular, certain waypoints functioned only as entry and exit waypoints to the targets that needed to be photographed, and thus only had a restriction on the effective radius to fly through. It was possible for Navigator/DEMPCs to refer to such waypoints by using the phrase “no restrictions” without mentioning speed or altitude explicitly, as in:

Next PRK has a radius of 2.5 and no restrictions

This kind of communication was also interpretable to the synthetic teammate. For the purposes of our analyses, we considered such communications to still be “full” communications. They were not analyzable for order in the same way as the communications that explicitly mention speed and altitude separately, but they did count toward addressing our research question about dropping or shortening of linguistic material (i.e., mentioning that there are no restrictions counts against the hypothesis that teammates may drop unused restrictions).

#### 2.5.3. “Partial” Communications

In the case of waypoints that did not have the full set of restrictions, Navigators/DEMPCs had the option to only mention the restrictions that did exist, and leave it implicit that the other restrictions did not apply. An example, without altitude included:

Next is HArea, radius is 5, speed is 50 to 200

Such communications were categorized as being “partial” communications. These communications provide a direct test of our hypothesis that Navigators/DEMPCs over time would drop unnecessary restrictions, following the more general pattern of “shortening” language that occurs as interlocutors work together on a joint task. Thus, we might expect to see Navigators/DEMPCs switching from full communications in the earlier missions to partial in the later missions. On the other hand, such communications could also be more challenging for teammates to interpret due to the requirement to assume a potential value (i.e., none) from the absence of the explicit mention in the communication. Further, if Navigator/DEMPCs have developed a particular routine for the ordering of the information (e.g., “speed,” “altitude,” “radius”), leaving any of the restrictions out would represent a deviation from that routine.

Sometimes also Navigator/DEMPCs would be asked to repeat a particular restriction for a particular waypoint, and these communications were also categorized as “partial” communications. For example:

Pilot/AVO: What's the speed again?
Navigator/DEMPC: 50 to 200

Such communications received the additional code “repeat.”

#### 2.5.4. Multiple Communications

Although mentioning a single waypoint and its restrictions within a single communication makes a nice “package” we also considered communications where this information was spread out across multiple communications. For communications to count as part of a “multi-communication,” they needed to be about a single waypoint, occur within close proximity to each other within the dialogue, and be about complementary information relative to the restrictions. For example:

Navigator/DEMPC: PRK has a radius of 2.5
Pilot/AVO: Any other restrictions?
Navigator/DEMPC: No speed or altitude

It was possible to have either “full” multi-communications (all three restrictions are mentioned across the multiple communications) or “partial” multi-communications (e.g., only speed and radius are mentioned, each in separate communications). Repeats of restrictions that had already been stated were always coded as being “partial” communications, and not part of a multi-communication.

The ability to disambiguate referents across multiple communications was not implemented in the synthetic teammate. Thus, multi-communications represent a deviation from what the synthetic teammate was capable of interpreting. Consequently, participants in the evaluation experiment were instructed to provide all waypoint information in a single text communication. It was an open question, however, to what extent the teams in the speech and text experiments might adopt such packaging. When speakers need to convey a large amount of information, it is common for speakers to provide the information in “installments.” For example, we have a conventionalized routine of providing phone numbers in installments (i.e., smaller groups of numbers) rather than providing the entire number all at once (Clark and Schaefer, 1987).

Table 3 summarizes the analyzed Navigator/DEMPC communication types explained above. Given the fact that these experiments were not originally designed to test our questions, many of the analyses were exploratory in nature. However, we did have some specific predictions. Given previous research on lexical and syntactic entrainment, we predicted that Navigator/DEMPCs would settle (possibly across the first four missions) into a specific packaging routine out of the multiple packaging options for the restrictions that they had. This routine would manifest more specifically as a particular ordering for the restrictions (when they were all mentioned). Different teams would likely converge on different orders however, similar to how different pairs adopt different terms in the Tangram task (Clark and Wilkes-Gibbs, 1986). As to whether or not the unused restrictions would be mentioned, there were a number of possibilities. One was that Navigator/DEMPCs might begin by always producing “full” communications, even for waypoints that did not have particular restrictions, and over time drop the unused restrictions (assuming that they were able to maintain common ground with the other teammates). It was also possible, however, that Navigator/DEMPCs might continually mention the unused restrictions because of having entrained on that particular packaging. With regard to mention, we might see differences between the teams in the speech experiment compared to the other two experiments where the teams communicated in text. Because the text environment imposes additional constraints that make a rapid back and forth exchange of information more difficult, Navigator/DEMPCs might produce more full communications compared to the speech group, given that it would be harder for the other teammates to ask for clarification about unmentioned restrictions, and also the chat interface being permanent would make it less likely they would need to repeat themselves. We might see faster convergence on an ordering for speed, altitude, and radius compared to the speech group. In addition, we might see these effects even more strongly in the evaluation experiment, and specifically the condition with the synthetic teammate pilot. Although the teammate was capable of interpreting a variety of packaging, it itself did not have entrainment capabilities, and the fact that it did not communicate in a completely human-like way might push Navigator/DEMPCs to more fully routinize their communications with regard to the waypoints (similar to how participants show greater alignment in syntactic structure when they think they are communicating with a computer; Branigan et al., 2003). More specifically, we might expect to see more “full” communications compared to “partial” communications, and also a faster convergence on a specific ordering for speed, altitude and radius. We expected to see hardly any multi-communications since all Navigator/DEMPCs in the evaluation experiment were specifically instructed not to use them, regardless of whether they interacted with a synthetic teammate pilot or not.

**Table 3:** Summary of analyzed Navigator/DEMPC communication types.

**Communication type**	**Speaker**	**Example**
Full canonical	Navigator/DEMPC	Next waypoint is FArea, the **speed** is 50 to 200, the **altitude** 500 to 2000, and the **radius** is 5
Full with “restrictions”	Navigator/DEMPC	Next PRK has a **radius** of 2.5 and **no restrictions**
Partial	Navigator/DEMPC	Next is HArea, **radius** is 5, **speed** is 50 to 200
Multi	Navigator/DEMPC*Pilot/AVO*Navigator/DEMPC	PRK has a **radius** of 2.5 *Any other restrictions?* No **speed** or **altitude**

## 3. Results

### 3.1. Full vs. Partial Communications

Because the analysis of the ordering of the restrictions was predicated on having a sufficient number of “full” communications with explicit mentions to analyze, we first wanted to see to what extent Navigator/DEMPCs produced full communications in comparison to partial communications, and whether this proportion changed across the first four missions. Recall that “full” communications constitute both communications that mention the full set of restrictions (speed, altitude, and radius) and also communications that mention the radius and “no restrictions” (refer to Table 3). We had predicted two possible outcomes. One was that Navigator/DEMPCs (perhaps over time) would drop or leave unmentioned any restrictions that were not relevant to a particular waypoint. Such an outcome would be in keeping with previous research that found conversational participants tend to shorten their communications over time as common ground is established and maintained. The second prediction was that Navigator/DEMPCs might continue to mention unused restrictions (perhaps particularly in the text groups where maintaining common ground and understanding was more difficult) because they had entrained on a particular packaging routine for discussing the waypoint restrictions which included all three restrictions (and thus would be disinclined to switch the packaging routine for different kinds of waypoints).

**Figure 2:**
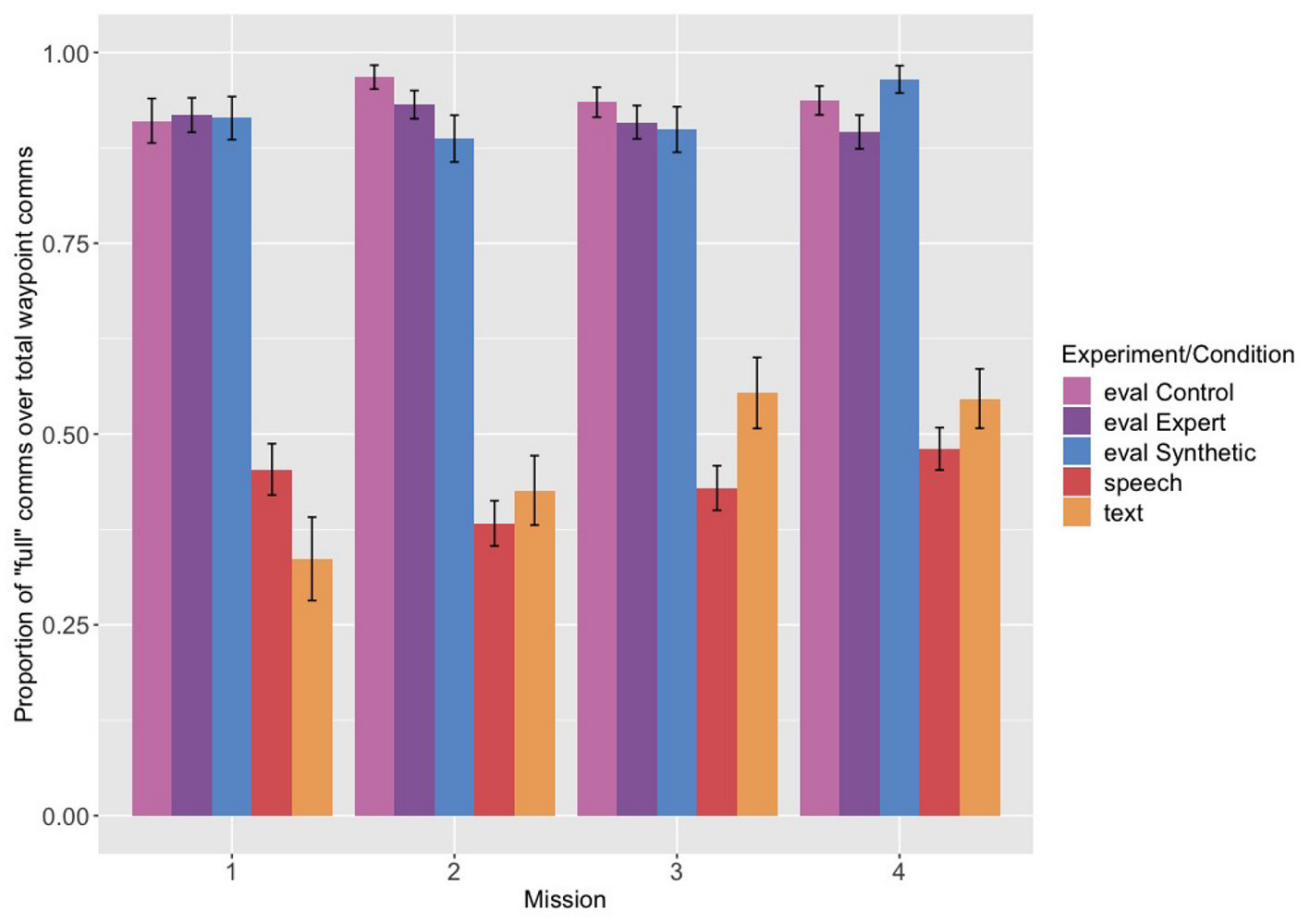
Proportion of “full” communications out of the total of full and partial communications, across the first four missions from each experiment. Eval refers to the evaluation experiment and its related conditions (control, expert pilot, and synthetic teammate pilot), speech to the speech only experiment, and text to the text experiment.

The proportion of full communications out of the total of full and partial communications across the first four missions is shown in Figure 2. Navigator/DEMPCs in the evaluation experiment, surprisingly regardless of condition, produced full communications far more often than partial communications (around 90%). The Navigator/DEMPCs in the speech experiment in comparison did not exclusively produce full communications; in fact they were produced only 40–50% of the time. The proportion of full communications also did not appear to decrease or increase across missions in the speech group. The high proportion of full communications in the evaluation experiment was not due to the text interface, however, as the text experiment showed a different pattern. Navigator/DEMPCs in the text group used a mix of full and partial communications like the speech group, but the relative proportion of full communications increased as they progressed through the first four missions. We can attribute this change to a couple of possible sources. One source is that the Navigator/DEMPCs may be converging on a packaging routine across missions, but rather than shortening or dropping information, they are converging on mentioning the restrictions more frequently. Another possibility is that in the later missions Navigator/DEMPCs received fewer requests to repeat the restrictions as the other teammates became accustomed to making use of the chat history (recall that in situations where Navigator/DEMPCs repeated only a subset of the restrictions, such communications were classified as partial communications). Removing communications that were labeled as “repeats” from the dataset resulted in an overall increase in the proportion of full communications across all the missions in the text group, but without changing the overall upward trend. The fact that the upward trend remains suggests that Navigator/DEMPCS in the text group were not merely dropping repeated mentions, but also shifting the proportion with which they used full communications as opposed to partial communications to talk about the waypoints. It is also not the case that Navigator/DEMPCs in the text experiment began navigating to more waypoints in the later missions that required mentioning all three restrictions compared to the earlier missions. The proportion of waypoints visited that required all restrictions remained comparable across all four missions (see Table 4 for a full breakdown of the number of waypoints requiring all restrictions vs. waypoints requiring partial restrictions for all experiments across the missions). It is notable that although the proportion of full communications increased in the text experiment, it never reached above 90% like in the evaluation experiment. The high proportion of full communications in the evaluation experiment is thus somewhat unexpected, particularly since it appears for all three conditions. We suspect that because Navigator/DEMPCs in the evaluation experiment were given the example of a single, full communication as part of their instructions for how to communicate if they had a synthetic pilot, that they may have been biased to overuse this type of packaging compared to how they would have used it without any kind of instruction on packaging.

**Table 4:** Total counts of communications broken down by whether the waypoint being mentioned required all restrictions or only some.

**Experiment**	**Mission**	**All restrictions**	**Partial restrictions**	**Prop all restrictions**
Speech	1	93	219	0.298
	2	115	214	0.350
	3	136	225	0.377
	4	160	229	0.411
Text	1	36	68	0.346
	2	50	72	0.410
	3	42	88	0.323
	4	72	100	0.419
Eval control	1	34	89	0.276
	2	47	108	0.303
	3	35	134	0.207
	4	57	134	0.298
Eval expert	1	53	130	0.290
	2	58	146	0.284
	3	57	162	0.260
	4	67	154	0.303
Eval synth	1	39	89	0.305
	2	44	89	0.331
	3	25	104	0.194
	4	45	97	0.317

We conducted a mixed-effects logistic regression with the probability of producing a full utterance as the dependent variable, and experiment and mission (continuous and centered) as the independent variables, with random intercepts by participant. For this analysis (and all mixed-effects analyses reported in the paper) we included the most complex version of the random effects that still allowed the model to converge. The speech experiment served as the reference group (full output of the model is provided in the Supplementary Materials). The coefficient comparing the evaluation experiment as a whole^1^ to the speech experiment was significant (*B* = 3.159, *p* < 0.05), but the coefficient comparing the text experiment to the speech experiment was not (*B* = 0.098, *p* = 0.8), suggesting that, on average across the missions, the speech and text groups produced a comparable proportion of full communications to partial communications. The mission coefficient for speech (the reference group) was not significant (*B* = 0.030, *p* = 0.6), nor was the mission by evaluation experiment interaction (*B* = 0.009, *p* = 0.9); however the mission by text experiment interaction was (*B* = 0.315, *p* < 0.05), confirming the positive increase in the proportion of full communications across the missions for the text group compared to the speech group.

### 3.2. Single vs. Multi-Communications

**Figure 3:**
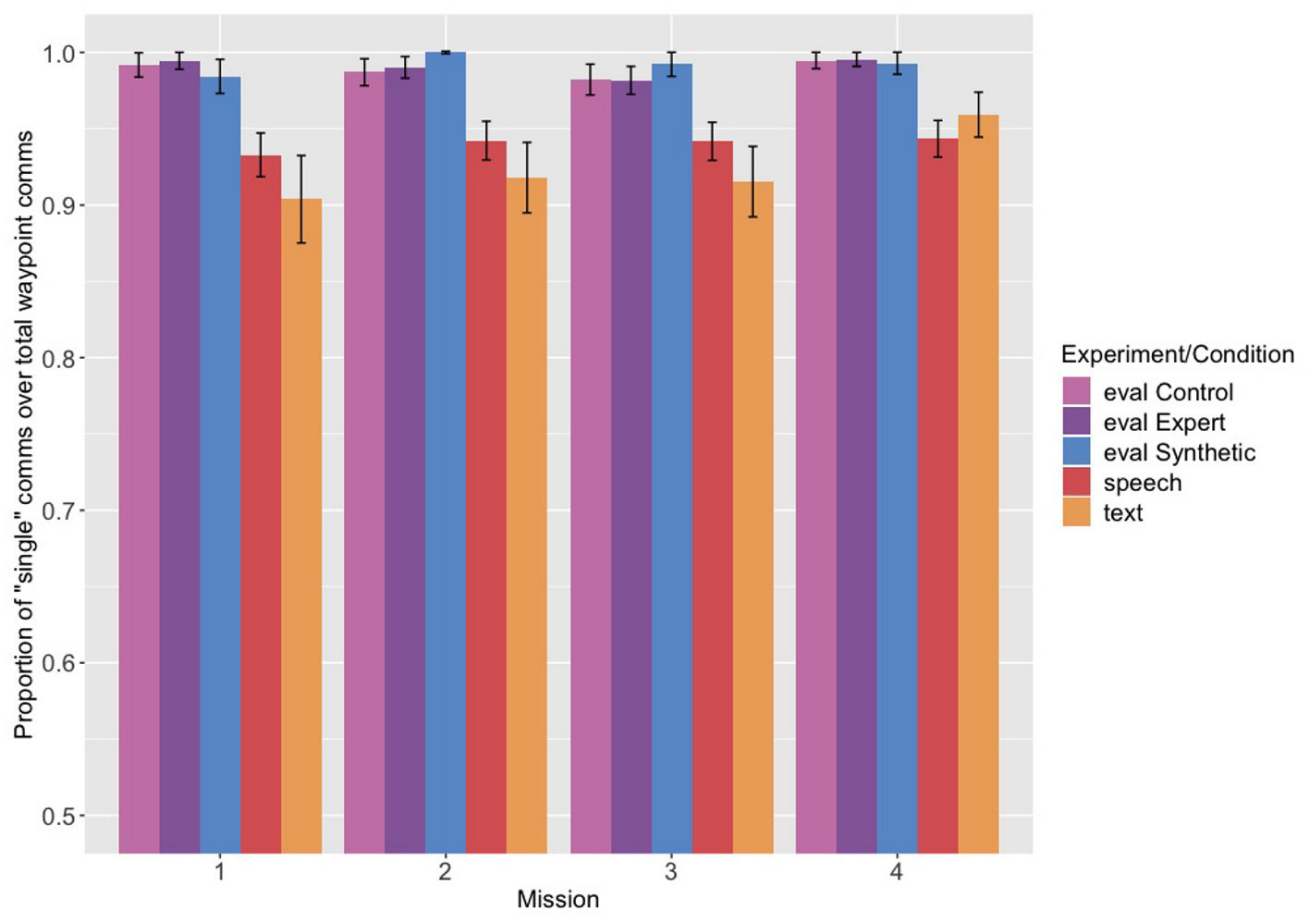
Proportion of “single” communications out of the total of single and multi-communications, across the first four missions from each experiment. Eval refers to the evaluation experiment and its related conditions (control, expert pilot, and synthetic teammate pilot), speech to the speech only experiment, and text to the text experiment.

Because we were also interested in whether restriction order would be maintained across multiple communications, we next compared the Navigator/DEMPCs' use of multi-communications compared to single communications to assess how frequently they were used. (The proportion of single communications out of the total of single and multi communications is shown in Figure 3). We predicted that if the Navigator/DEMPCs packaged the restrictions across multiple communications, they would be more likely to do so in the speech and text experiments, since they were instructed not to use them in the evaluation experiment. In fact, though all groups used predominantly single communications, the speech and text groups did use proportionately more multi-communications compared to the evaluation groups. The use of multi-communications did not appear to change across missions.

We conducted a logistic regression with the probability of producing a single communication as the dependent variable, and experiment and mission (continuous and centered) as the predictors, with random intercepts by participant. The speech experiment was again the reference group. We found a significant difference between the evaluation experiment as a whole compared to the speech experiment (*B* = 2.252, *p* < 0.05) and no significant difference between the speech and text experiments (*B* = 0.150, *p* = 0.8). The mission coefficient was not significant, nor were any of the interactions (full output of the model is reported in the Supplementary Materials). Although we found a significant difference between the evaluation experiment and the speech experiment, we feel it is important to note that the use of multi-communications does not appear to be a routine that is frequently employed by Navigator/DEMPCs either in the speech experiment or the text experiment. The fact that Navigator/DEMPCs cannot use multi-communications with the synthetic teammate pilot may not therefore have a huge impact on their ability to communicate and perform the task. Because multi-communications represented such a small proportion of communications across all of the experiments, we collapsed across single and multi-communications for our analyses of the ordering of the restrictions.

### 3.3. Ordering of Restrictions

**Figure 4:**
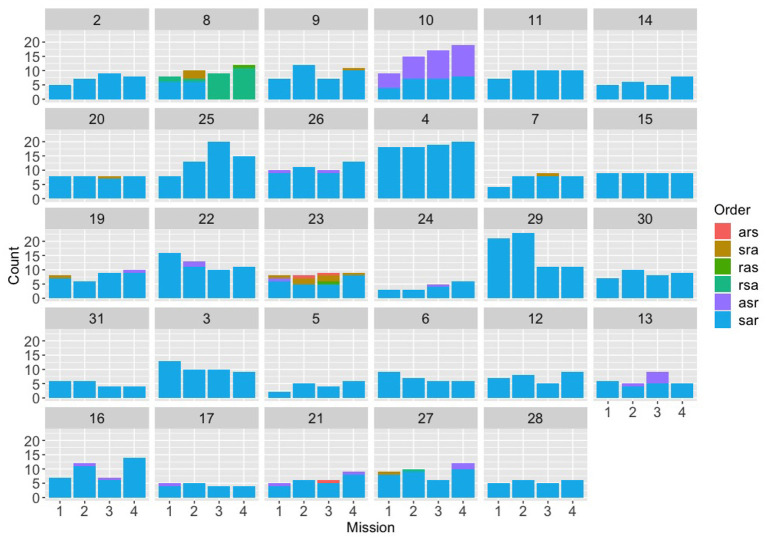
Counts for each of the six possible orders for (s)peed, (a)ltitude, and (r)adius, across the first four missions for the evaluation experiment, broken down by team. The first nine teams (2-26) were in the control condition; the next 10 teams (4-31) were in the expert condition; the last 10 teams (3-28) were in the synthetic teammate condition.

**Figure 5:**
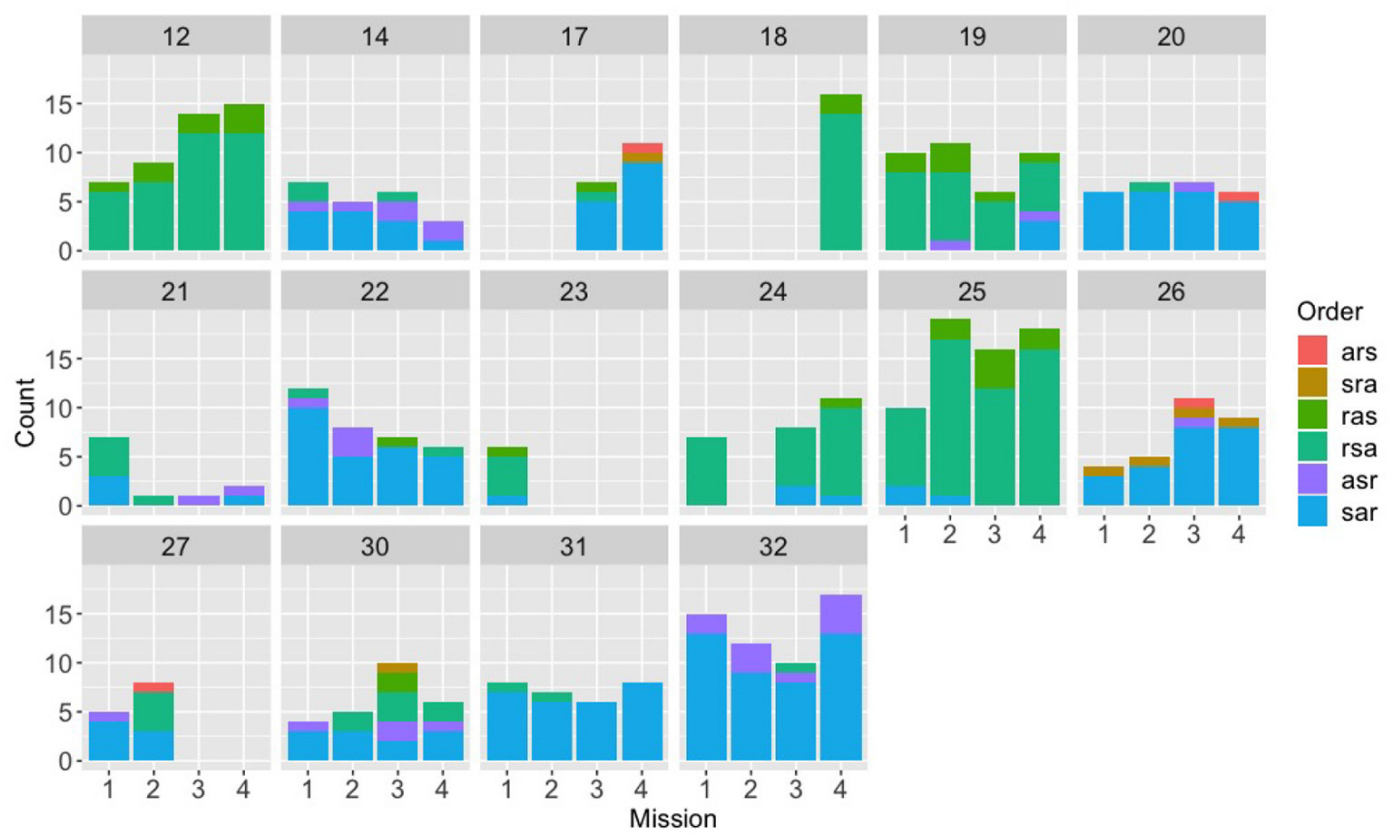
Counts for each of the six possible orders for (s)peed, (a)ltitude, and (r)adius, across the first four missions for the speech experiment, broken down by team.

**Figure 6:**
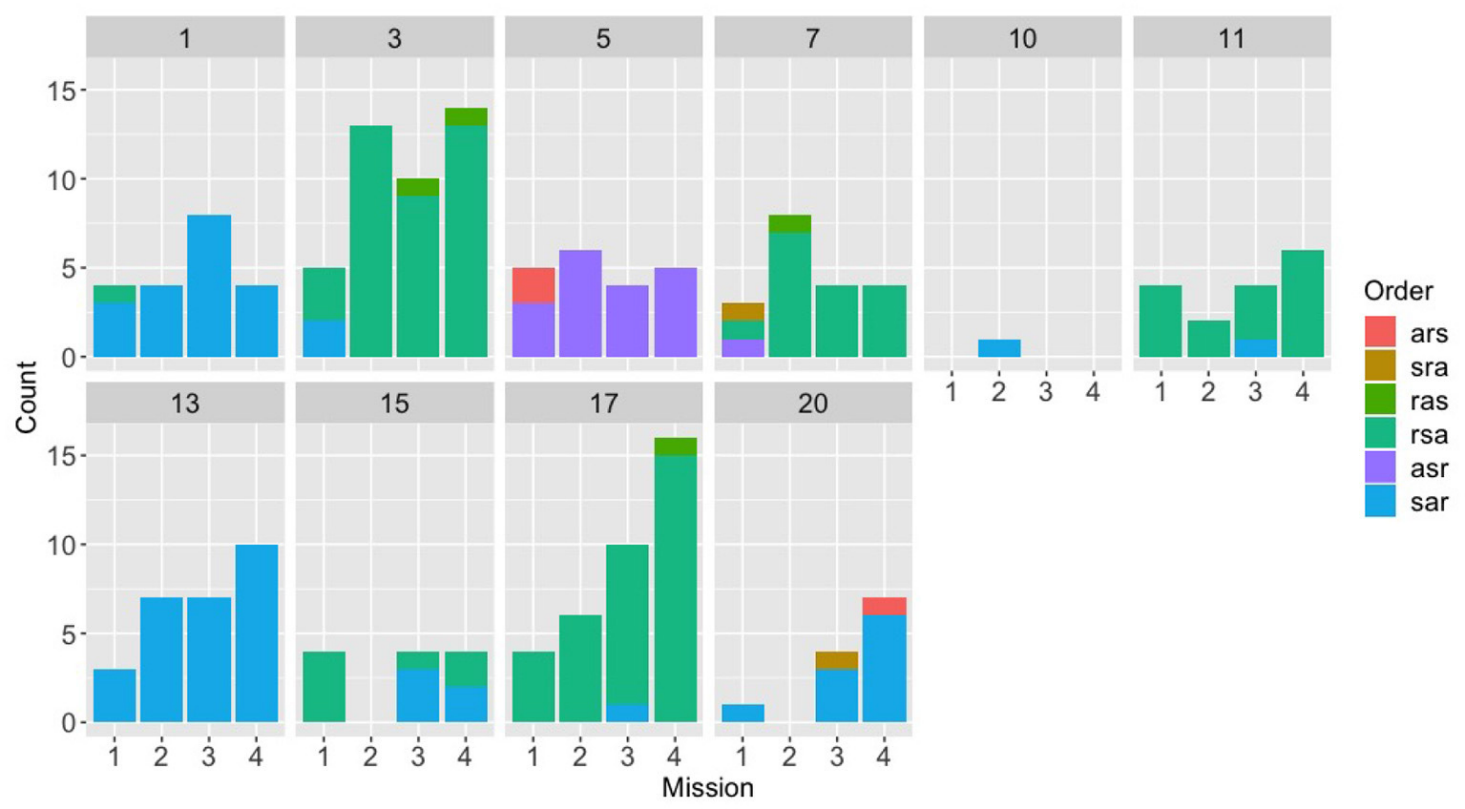
Counts for each of the six possible orders for (s)peed, (a)ltitude, and (r)adius, across the first four missions for the text experiment, broken down by team.

We next turned our attention to the full “canonical” communications (ones that mentioned explicitly speed, altitude, and radius separately) to investigate whether each team converged on a particular ordering for the three restrictions (and also whether different teams converged on different orders). We had predicted that if Navigator/DEMPCs did converge, different Navigator/DEMPCs from different teams might choose different orders. We had also predicted that convergence might be stronger in the evaluation experiment (particularly in the synthetic teammate condition) and also in the text experiment compared to the speech experiment due to the additional challenges of maintaining common ground in a text medium compared to the speech medium. Figures 4–6 plot the counts of different orderings of speed, altitude, and radius across the first four missions (where data were available) for each of the individual teams within the three experiments (evaluation, speech, and text). The first thing to note is that within the evaluation experiment, not only did many of the teams appear to have a preferred order, but they almost all appear to have used the same order (specifically “speed, altitude, radius”). Although the teams were not instructed to use a particular order, we suspect that the example used in the packaging instructions given to these Navigator/DEMPCs biased them toward using the ordering of “speed, altitude, radius.”

Looking at the speech group in comparison (Figure 5), a different picture emerges. The speech Navigator/DEMPCs, by and large, also appear to have chosen particular orders, but we see greater variety in which orders they have picked. This result supports our initial hypothesis that if Navigator/DEMPCs did converge on a particular ordering, they would not necessarily all converge on the same order. The same appears to be true of the Navigator/DEMPCs in the text group (Figure 6).

We had predicted that the Navigator/DEMPCs might converge on a particular order over time, across the missions. However, it appears to be the case that many Navigator/DEMPCs choose a preferred order during the very first mission and continued to use that order throughout. The low data counts per mission for some of the teams precluded us from conducting any statistical analyses to evaluate convergence (or lack of convergence) on a team-by-team basis; however we were able to evaluate the average convergence of teams within and across experiments by using recurrence quantification analyses.

We applied discrete auto-recurrence (ARQA) within individual teams, discrete cross-recurrence analysis (CRQA) between all pairs of teams within a given experiment, and CRQA between all pairs of teams across all experiments. Note that ARQA refers to recurrence analysis performed on a single time series of data (compared against itself); in this research, we use it to look at the sequence of communications within a single team. CRQA refers to recurrence analysis comparing two time series to each other; in this research, we use CRQA to compare the communication sequences between pairs of different teams. For each team pairing in CRQA, communications data were matched by mission, such that only communications from mission one were compared to mission one, mission two compared to mission two, and so on. Note that Team 10 from the text experiment was excluded from the RQA analyses because they only had one full communication instance in their data, which is not enough data for RQA. Because there were six possible orderings for the restrictions of speed, altitude, and radius, recurrence was defined for each instance as using the same ordering option within those six choices. Analyses were run in R leveraging the *crqa* package (Coco and Dale, 2014), with embedding dimension variable set to 1 and rescale and normalize parameters set to 0. The minimum length for a diagonal line was set to 2.

The preference for a particular ordering for the restrictions might emerge in two ways. One, convergence may emerge slowly over the experiment; teams may try multiple orderings and select one over time. The dynamics for this pattern would reflect some exploration early (low recurrence for early trials) and then emergence of more recurrence for later trials, reflected in lower recurrence or recurrence points that do not form diagonal or vertical structures. Two, convergence may emerge quickly with teams selecting an ordering early and sticking with that ordering throughout. The dynamics of this pattern would be captured by long vertical structures (laminar structures) in the recurrence plots. Laminar structures reflect recurrence of the same state over time. To examine evidence for these possible dynamics, we looked at the recurrence rates and mean vertical line lengths (called the trapping time statistic) in all ARQA and CRQA.

**Figure 7:**
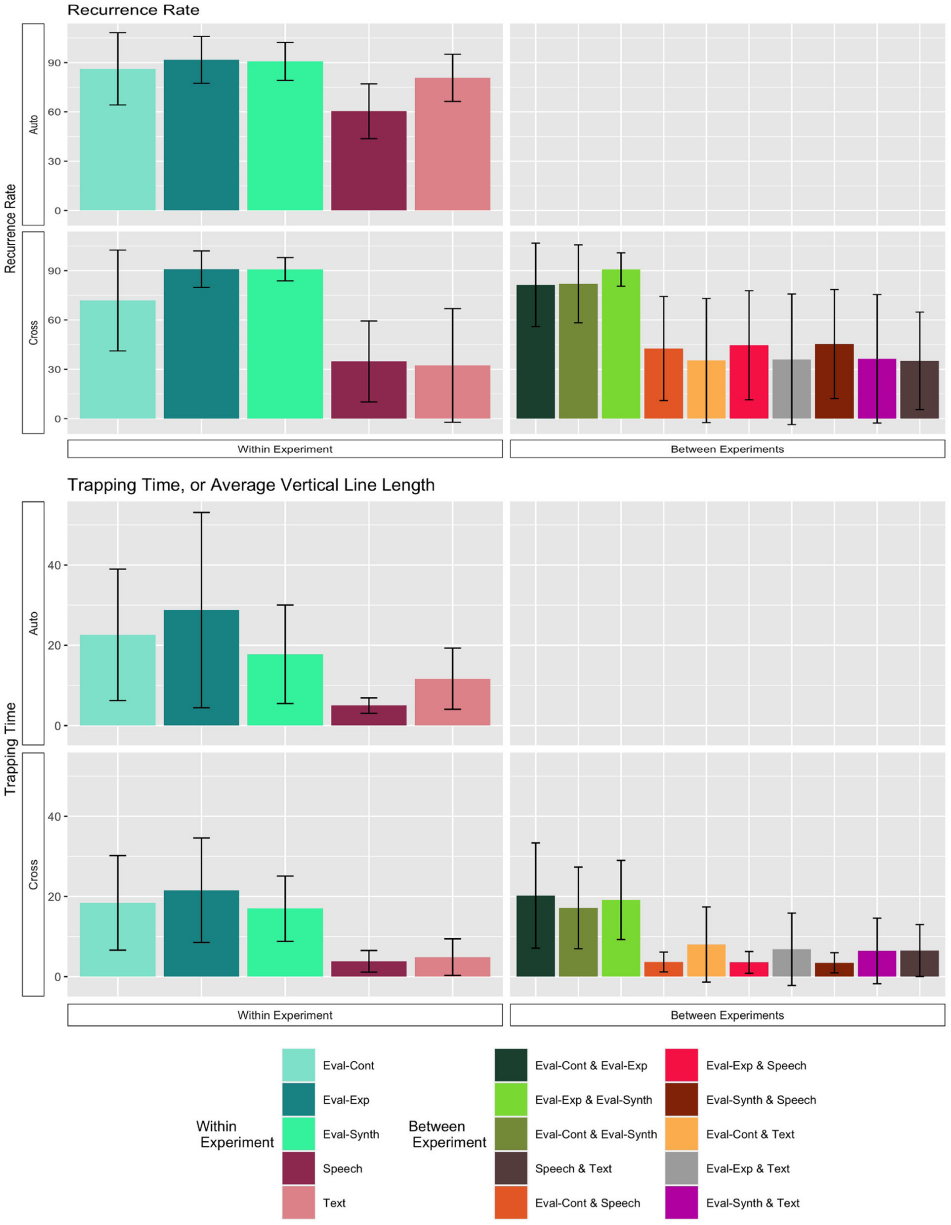
Recurrence rate and trapping time statistics for RQA. The upper plots give the Auto-RQA statistics, and the bottom rows give the Cross-RQA, split by within (left) and between (right) Experiment conditions. Bars give the mean statistics; error bars are ±1 standard deviation of the mean.

Recurrence rate and trapping time statistics for both the ARQA (upper left quadrant) and CRQA (lower row) analyses are shown in Figure 7. From the plots, we can observe that the three evaluation experiment conditions are higher both for ARQA and for CRQA, and both for the within and between experiment comparisons, compared to the speech and text experiments.

To further explore the trends in the plots, we conducted a series of analyses. We first conducted two-way analysis of variance (ANOVA) on recurrence rate with the between subjects factor *experiment* (5 levels: control, expert, synthetic, speech, text) and the between subjects factor recurrence *type* (2 levels: Auto-Recurrence, Cross-Recurrence). We evaluated results relative to the null hypotheses of no difference between groups, and used the Type I error rate α = 0.05. Visually, we are comparing only the data represented by the left-hand side of the plots of Figure 7. For recurrence rate, there was a significant main effect of experiment [*F*_(4, 322)_ = 94.64, *p* < 0.001], a significant main effect of recurrence type [*F*_(1, 322)_ = 29.01, *p* < 0.001], and a significant interaction of experiment and recurrence type [*F*_(1, 322)_ = 6.19, *p* < 0.001]. *Post-hoc* Tukey HSD (*p*-value adjusted for number of comparisons) comparisons confirmed that overall, ARQA recurrence rates were higher than CRQA recurrence rates (*p*_*adj*_ < 0.001). Within the ARQA results, using *post-hoc* Tukey HSD paired comparisons, the mean recurrence rate for speech was trending lower than the control experiment (*p*_*adj*_ = 0.16), expert experiment (*p*_*adj*_ = 0.02), and synthetic experiment (*p*_*adj*_ = 0.03). Within the CRQA results, there was no difference between speech and text mean recurrence rates, and no difference between expert and synthetic evaluation experiments. The control condition had lower CRQA recurrence rate than both the expert (*p*_*adj*_ = 0.01) and the synthetic (*p*_*adj*_ = 0.01) conditions. Speech and text had lower cross-recurrence rates than all evaluation experiment conditions (all pairwise comparisons *p*_*adJ*_ < 0.001). Speech and text also had lower CRQA rates compared to their ARQA rates (speech *p*_*adj*_ < 0.001; text *p*_*adj*_ < 0.001). The comparable comparisons for all the evaluation experiment conditions were not significant (control *p*_*adj*_ = 0.8; expert *p*_*adj*_ = 1; synthetic *p*_*adj*_ = 1).

We then conducted a similar two-way ANOVA on the trapping time statistics for the within-experiment analyses. For trapping time, there was a significant effect of experiment [*F*_(4, 322)_ = 55.03, *p* < 0.001], and significant effect for recurrence type [*F*_(1, 322)_ = 6.48, *p* = 0.01]. There was not an experiment x recurrence interaction for trapping time [*F*_(4, 322)_ = 0.945, *p* = 0.44).

*Post-hoc* Tukey HSD pairwise comparisons (*p*-values adjusted), indicate that trapping time for ARQA was significantly larger than CRQA trapping time (*p*_*adj*_ = 0.012). For the experiment conditions, pairwise comparisons indicate that speech and text are consistently lower than all evaluation experiment conditions (all *p*_*adj*_ < 0.001), and there is no statistical difference between speech and text (*p*_*adj*_ = 0.82), or between expert and synthetic (*p*_*adj*_ = 0.99). The control condition had consistently lower trapping time than the expert (*p*_*adj*_ < 0.004) and synthetic (*p*_*adj*_ < 0.004) conditions.

Taken together, the ARQA statistics support that individual Navigator/DEMPCs do repeatedly use orderings (recurrence rate) and in particular a preferred ordering (trapping time), though to different degrees if we compare between experiments. Both statistics are higher in the evaluation experiment conditions than in the speech experiment. For recurrence rate, the text condition is higher than speech, closer to the evaluation experiments; the text experiment was more similar to the speech experiment for trapping time. This pattern indicates that in the non-speech conditions, Navigator/DEMPCs exhibited higher amounts of repeating the same orderings, but only in the evaluation experiments did we see high trapping time, or stronger evidence of teams selecting a preferred ordering and sticking with it throughout the missions.

We note however that the mean trapping time values in Figure 7 are longer than the numbers of repetitions per mission in Figures 4–6. This provides evidence that teams in all experiments were sticking with their preferred orderings for periods longer than a single mission, or put differently, that they were carrying their preferences between missions.

Additionally, if navigators had randomly selected different orderings, then we would expect recurrence rates and trapping time statistics close to zero. However, both the ARQA and CRQA statistics show evidence that there are repeated, preferred orderings in all the conditions as exhibited by the mean statistics and standard deviation bars above zero. The recurrence rate and trapping time are generally higher, both in repetition and convergence on a single preferred ordering, in the evaluation experiment conditions, however. Note also that the ordering preferred may differ between teams; ARQA does not indicate which is the preferred ordering, just some quantification of how much a single ordering is preferred as indicated by the longer trapping time. In fact, that the CRQA rates were significantly lower than the ARQA rates for speech and text experiments suggests that different Navigator/DEMPCs did chose different orderings in those experiments, hence why the recurrence rate was lower comparing between Navigator/DEMPs than within a single Navigator/DEMPC.

We next considered the between-teams comparisons of ordering usage with CRQA, which were not included in the two-way ANOVAs as they fall outside the factorial design combinations in that analysis. Here we compare all possible pairing of teams, both teams within a single experiment condition and then between experimental conditions. The lower row for each statistic in Figure 7 shows the statistics for the CRQA, with the within-experiment CRQA on the left and the between-experiment CRQA on the right. Mean values are higher for CRQA recurrence rate and CRQA trapping time, for the pairings of teams within all the evaluation experiment conditions (bars in the six green shades). Thus, parings within the control, expert, and synthetic evaluation conditions, and the pairing between these evaluations conditions, show higher amounts of recurrence overall than pairings within the speech and text experiments, higher than the speech-text pairings, and higher than pairings bewteen the eval-and-speech and eval-and-text pairings. This means that evaluation experiment Navigator/DEMPCs compared with each other showed a higher amount of cross-recurrence or repetition of the restriction orderings even between the control-expert-synthetic conditions. We can interpret this result as indicating that the evaluation experiment Navigator/DEMPCs tended to use the same restriction orderings even if on different teams. The similar trend of longer cross-recurrence trapping times in the evaluation experiment implies that those longer periods of recurrence are dominated by long sequences that repeat the same state, showing similar preferred use of a single restriction ordering between teams. On the other hand, the low cross-recurrence and low cross trapping time in the other pairings suggests that teams were not showing similar preferences or similar repetition of orderings. Even if individual teams had higher auto-recurrence rates or auto-trapping times, the preferences with the team that were repeated were not similar to the preferences or usage patterns of other teams.

In all, the RQA statistics provide evidence that the Navigator/DEMPCs in the evaluation experiment do settle on a preferred order and tend to stick with it more than the speech and text experiment Navigator/DEMPCs. Because the low data counts precluded us from analyzing individual teams for convergence on a particular ordering, these results do not imply that individual Navigator/DEMPCs in the speech and text experiments had *no* preferred ordering for the restrictions, only that on average, the Navigator/DEMPCs in the speech and text experiments exhibited more variability than Navigator/DEMPCs in the evaluation experiment both within themselves and between teams.

### 3.4. Team Performance

**Figure 8:**
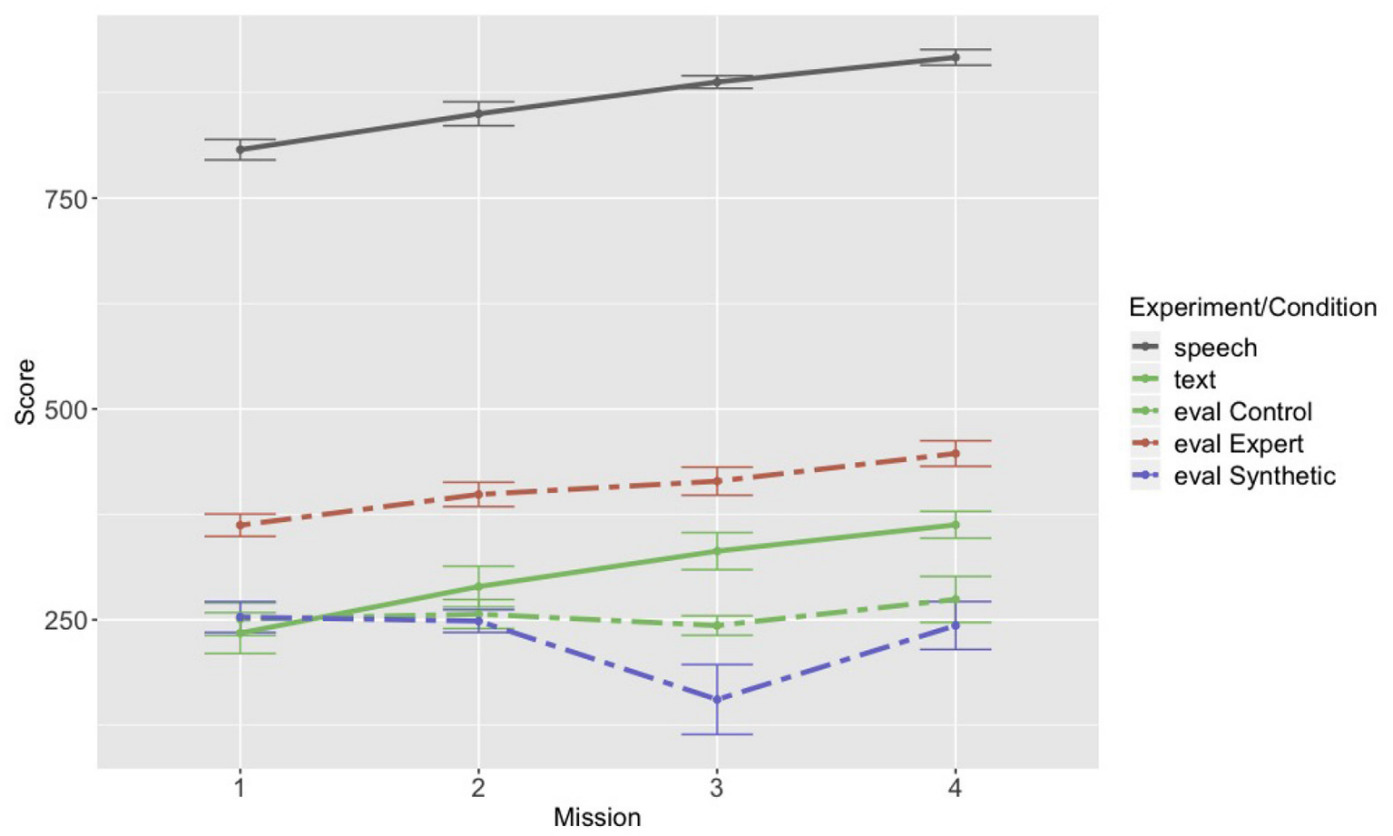
Performance across the first four missions for each of the text and speech experiments and the evaluation experiment conditions: control, expert, and synthetic.

Thus far, we have demonstrated that there are notable differences between the language packaging of the evaluation groups and the speech and text groups. Might such differences in packaging have consequences for performance? We revisited the performance metric collected from the original three experiments (described in section 2.1). Figure 8 plots the performance metric across the first four missions for each experimental condition. The first thing to note is that the speech group performed noticeably better than any of the other groups. This difference we attribute to the fact that it is easier to simultaneously communicate in speech and operate a visual display than it is to communicate through a chat interface (visual) and operate a visual display. Second, the expert group in the evaluation experiment performed better than any of the other remaining groups, including the original text group. However, both the expert group and the original text group showed an improvement in performance across the first four missions, which the control group and the synthetic pilot group did not. A mixed-effects linear regression confirmed our observations from the graph: with speech as the reference group, we used score as the dependent variable and mission (continuous and centered) and experiment/condition as independent variables, with random intercepts by team. The coefficient comparing mission for the control group compared to the speech group was negative and significant (*B* = –26.305, *p* < 0.05), and the coefficient comparing mission for the synthetic group to the speech group was also negative and significant (*B* = –41.008, *p* < 0.05; full model output provided in the Supplementary Materials). We also re-ran the analysis with the text experiment as the reference group to fully confirm what appeared to be a difference between the original text group and the control group, and the coefficient comparing the change in performance across missions for the control group to the text group remained significant even with an adjusted *p*-value (*B* = –37.077, *p* < 0.025). We hypothesize that the difference in performance between control group and the original text group (which are the two most comparable groups) may be due to the instructions they were given with regard to packaging of language. The original text group was not given any instructions as to how to package the restrictions. The control group was given instructions that appear to have biased them toward producing an unnaturally high proportion of full communications, with hardly any variability in order across teams. Although the use of a full, single communications is a reasonable package that all of the teams use, if it is not developed as a routine during the team's natural interaction, it may have a detrimental effect on overall team performance.

## 4. Discussion

We found evidence that, similar to lexical and syntactic entrainment found in previous research, teams do reuse specific orders more than others, and that they do not necessarily pick the same order as other teams. The evaluation experiment groups showed surprisingly little variability in their orders, which we suspect was due to the instructions that these teams were given (even though they were not told that they needed to use a specific order, nor that they had to produce “full” communications). Low data counts in the individual missions for many of the teams made it difficult to determine the time frame for which individual teams may settle on an order; many Navigator/DEMPCs seemed to stick to a preferred order just within the first mission, rather than having an order come to dominate in the later missions. One possibility is that selection of a particular order by the Navigator/DEMPC might be too rapid for us to detect. Isaacs and Clark (1987) found that experts and novices seem to be able to converge on each other's level of understanding within the exchange of only two utterances, and adapt their language use accordingly there after. Another possibility is that the structure of the task already biases the Navigators/DEMPCs toward certain orders, which should also make fully free variation between the orders less likely. Similar results were found in the commonly used Maze task (Garrod and Anderson, 1987), where, despite finding the full range of possible organizing schemes, the researchers found that particular schemes seemed to be more common across teams, and they observed even greater convergence onto a single scheme when members of different pairs had to interact with each other across games (Garrod and Doherty, 1994). It is also unclear to what extent Navigator/DEMPCs switching between orders, even when they seem to have one preferred order, should be considered “noise.” Also in the Maze task, Garrod and Anderson (1987) found that pairs rarely stuck to only a single scheme for the entirety of the experiment, even though pairs clearly had preferences for a subset of all the possible schemes.

We also found that both the speech and the text groups produced on average roughly equal proportions of “full” communications (explicitly mentioning speed, altitude, and radius, or “no restrictions” for speed and altitude combined) and “partial” communications, though the text group showed a significant upward trend in the proportion of full communications across the first four missions. This result suggests that in the text group there may have been additional pressure to use the same full packaging, regardless of whether the waypoint had all of the restrictions or not. We did not find evidence of the groups producing proportionately fewer full communications over time, arguing against an alternative prediction that Navigator/DEMPCs might start by producing full communications and later shorten them by dropping the unused restrictions as they advanced in the task. The evaluation groups produced an unnaturally high proportion of full communications, which we hypothesize was due to the instructions they received with regard to communicating in the task. We also found that Navigator/DEMPCs in the speech and text experiments were more likely to produce “multi-communications” compared to the Navigator/DEMPCs in the evaluation experiment; however they still produced predominantly single communications, suggesting that not having the option to produce multi-communications may not cause much of a hindrance to team performance.

The differences in packaging of information, namely the option to choose different orders for speed, altitude, and radius, and also the option to produce partial communications along with full communications, may have contributed to the fact that teams in the text experiment show improved performance across the missions, whereas the comparable group in the evaluation experiment (i.e., the control group) did not. The fact that the expert group in the evaluation experiment showed improved performance is perhaps not surprising; expert knowledge of the task surely contributes to better overall performance for a team, and this knowledge seems to be enough to overcome whatever hindrance may be caused by the language packaging instructions. It is unclear, however, to what extent it may be more feasible to develop a synthetic teammate that has the capabilities of the expert pilot in the expert group, as opposed to a synthetic teammate that is more like a “naïve” pilot but is capable of flexibly adapting its language routines or participating in entrainment. The fact that the Navigator/DEMPCs in the evaluation experiment unnecessarily restricted their information packaging compared to what the teammate was capable of understanding is unfortunate, though provides a good example of what the potential effects of such restrictions might be. These results also speak to the difficulties in getting human teammates to accurately understand what a synthetic teammate's capabilities truly are, so that human teammates neither over- nor under-estimate the teammate's abilities.

As research continues to explore how to develop synthetic teammates that can properly situate language within a given context (e.g., Bonial et al., 2020), recognize and eventually learn new vocabulary (e.g., Scheutz et al., 2019), and more generally become more flexible and human-like in their use of language, it will be important to establish whether or not it will be beneficial for teammates to entrain on particular linguistic structures, and if so, which structures. Our results suggest that information packaging may be a good candidate to consider for synthetic teammate entrainment, though further research is needed. Ordering of information in particular may become routinized similar to lexical entrainment, but the routinizing of mention is more complex, and potentially different depending on the communication medium. We also found evidence that artificially inducing entrainment (particularly to an extreme degree) may have negative consequences for team performance. This result is consistent with research suggesting that the alignment of language is not always predictive of team performance (e.g., Rothwell et al., 2017), nor is it necessarily the dominant process in dialogue (e.g., Mills, 2014).

### 4.1. Insights for Synthetic Teammate Development

More often than not, synthetic teammates will be required to communicate with team members using language. It should come as no surprise that machines are not equipped to handle unfettered natural language. Indeed, the instructions to human participants in the evaluation experiment to package all waypoint information into a single text communication was provided to human participants to avoid pitfalls of reference disambiguation within the synthetic teammate. What did come as a surprise was that the presence of the communication instructions seems to be related to observed performance increases for teams free to adapt their linguistic constructions with experience. Given these results and the current lack of a complete natural language processing/understanding capability available to intelligent machines, what are the paths forward for the development of synthetic teammates communicating through language?

One path forward is for intelligent systems to operate over controlled languages (c.f., Kuhn, 2014). A controlled language is one that has a finite set of constructions in the targeted language (e.g., English), such as the Attempto Controlled English (ACE) controlled language (Fuchs, 2018). The benefit these systems provide is the ability to decompose communicated information into structures more easily processed by intelligent machines, such as the Attempto Processing Engine (APE). For example, APE translates text written in ACE into a *discourse representation structure*, providing a syntactical variant of first-order logic for further processing.

Another approach is to adopt human-animal teams as an example paradigm of how human-machine teams should interact and the potential benefits from its adoption (Phillips et al., 2016). Potential benefits include improvements to system design and trust. Similar to the controlled language approach, machines developed following the human-animal paradigm would also require a restricted lexicon and set of natural language constructions used for communication, as this is typically the case in human-animal teams. While each of these approaches is beneficial to machines' understanding of communications, they do not solve issues associated with human-machine mutual adaptation to each other's communications. What may be critical, and requires further research to determine, is if team members require the opportunity/capacity to adapt linguistic behaviors to other team members to further improve team performance with experience. Indeed, enabling machines to adequately communicate and coordinate with human team members may well require a combination of a controlled language approach within the human-animal team paradigm with machine capabilities to adapt to communication styles of different team members.

### 4.2. Further Considerations and Future Directions

Because this was a reanalysis of pre-existing data, there were a number of complications due to the structure of the task that are important to consider with regard to how they may have impacted our findings. One is that the number of waypoints a team hit during a mission was entirely dependent on the team itself. The map was open, and Navigator/DEMPCs were free to plot their own courses and determine how many waypoints they wanted to hit. If they encountered issues, they might not hit all of the waypoints on their route. The number of waypoints the team navigated to also determined how many waypoints the Navigator/DEMPC talked about, and consequently how many data points we had to analyze. Teams that struggled and hit fewer waypoints would also mention fewer waypoints. In future research we would use a task where each team produces closer to the same number of data points.

Another consideration is that the restrictions themselves conceptually were not “equal,” and this may have biased Navigator/DEMPCs to prefer certain orders over others. There appeared to be a preference across the Navigator/DEMPCs to group speed and altitude together (most commonly speed before altitude, but some teams also used altitude before speed). It was very rare though for Navigator/DEMPCs to use an order where radius came between speed and altitude. Both the speed and altitude restrictions apply to the aircraft itself (and are adjusted on the aircraft by the Pilot/AVO), whereas the radius has to do with how close the aircraft is to the waypoint (so the issue is more of timing). In future research, we would design a task where the information that needs to be ordered would be more “equal” so that we could get a better sense for how much variety is possible (and how fast convergence would be under such circumstances).

It is also important to consider the asymmetric nature of the roles of the teammates. In other kinds of tasks that have investigated common ground in conversation (e.g., the Maze task), the roles of the participants are more similar. Even in the Tangram task which is asymmetric because one person serves as the Director and the other as the Matcher, both participants often contribute to the descriptions and names of the cards to complete the task. In the RPAS task, only the Navigator/DEMPC ever needs to mention all three restrictions at the same time, which means that we could not look for repetitions of orders across different teammates (whereas repetition of names and descriptions does sometimes occur across participants in the Tangram task). In future work, we would like to use a task where all participants have need to mention all of the pieces of information, so that we can track ordering of information across them all.

There were also a variety of communications which the Navigator/DEMPCs produced that did not fall into the categories described in section 2.5 and therefore were not the subject of our current analyses. Such communications should be considered in future work however. For example, for the entry and exit waypoints that only had an effective radius restriction (which was always 2.5 mi), sometimes the Navigator/DEMPCs would not mention the restrictions at all, as in:

Next is PRK and it's an entry

This type of communication leaves it entirely up to the Pilot/AVO to recall that entry waypoints have an effective radius of 2.5 and no other restrictions. We only considered communications that mentioned at least one restriction for our analyses.

Also, because we focused our analyses on the communications from the Navigator/DEMPCs rather than the other teammates, we also did not analyze communications that, conceivably, were about the waypoint restrictions but contained no mention of the restriction by the Navigator/DEMPC. For example, in the following exchange:

Navigator/DEMPC: PRK is next
Pilot/AVO: Radius 5?
Navigator/DEMPC: Correct

it is the Pilot/AVO that makes mention of the restriction, and the Navigator/DEMPC merely provides confirmation. In future work, we plan to analyze this kind of collaborative exchange as a unit, which pairs may reuse across trials.

We also left un-analyzed communications where Navigators/DEMPCs mention multiple waypoints but only a single set of restrictions. For example:

Next are OAK and PRK, radius 2.5 for both

was counted as only a single data point, however there are technically two waypoints being mentioned at the same time. These kinds of parallel mentions should be considered in future work. Also, Navigator/DEMPCs, particularly in the speech data set, did sometimes mention multiple waypoints within a single push-to-talk comm, as in:

Next is HArea, speed 50 to 200 and radius 5, and then we have FArea, speed 50 to 200, altitude 500 to 1000, and radius 5

but such instances were counted as if they were two separate communications. Tracking such long groupings of multiple waypoints should be considered in future research since it represents potentially an even farther deviation from what should be considered “efficient” for the Navigator/DEMPCs to produce.

### 4.3. Conclusions

We sought to investigate whether Navigator/DEMPCs in a three person simulated piloting task would converge on particular routines for the *packaging* of restrictions related to waypoints. We also investigated whether these processes might be impacted by communicating through text chat rather than a push-to-talk voice interface, and whether the possibility of interacting with a synthetic teammate as the pilot further altered such processes. We found that, in general, Navigator/DEMPCs preferred a particular ordering for the waypoint restrictions, though only in the speech and text experiments did we see that different Navigator/DEMPCs choose different orders. Navigator/DEMPCs in the speech and text experiments were also more likely to produce “partial” communications compared to Navigator/DEMPCs in the evaluation experiment, though Navigator/DEMPCs in the text group increased in their proportion of “full” communications over the first four missions whereas the speech group did not. The rigidity in the communications of the evaluation experiment Navigator/DEMPCs may have been due to the fact that they were given instructions with regard to linguistic packaging that included specific examples which they emulated; this rigidity may have contributed to a lack of improvement in performance across the missions, even in the control teams who did not have to communicate with a synthetic pilot. These results suggest that teams do potentially entrain on the ordering of information similar to entrainment of other linguistic structures, and that interference with the natural process of convergence may hurt team performance. Future work will investigate to what extent such ordering routines may appear in other kinds of tasks, and thus whether or not such entrainment may be a beneficial addition to current synthetic teammate capabilities.

## Data Availability Statement

The data analyzed in this study is subject to the following licenses/restrictions: the data were originally collected and de-identified at a separate laboratory at Arizona State University; neither the ASU nor AFRL IRB have approved the data for public release. Requests to access these datasets should be directed to Christopher W. Myers, christopher.myers.29@us.af.mil.

## Ethics Statement

The studies involving human participants were reviewed and approved by Air Force Research Laboratory IRB. The patients/participants provided their written informed consent to participate in this study.

## Author Contributions

SB re-coded the data for the new analyses, worked on the new analyses, wrote the intro, data coding, results, and discussion sections. LB conducted specifically the ARQA and CRQA analyses, wrote that section of the results, and edited other sections of the paper. CM contributed the existing dataset, worked on the new analyses, and wrote the methods section and edited other sections of the paper. All authors contributed to the article and approved the submitted version.

## Conflict of Interest

The authors declare that the research was conducted in the absence of any commercial or financial relationships that could be construed as a potential conflict of interest.

## Footnotes

^1^A model that included coefficients for each condition within the evaluation experiment separately did not converge.
