# Supplementary figures and images for: How Packaging of Information in Conversation Is Impacted by Communication Medium and Restrictions
Source: Front Psychol. 2021 Apr 16;12:594255. doi: 10.3389/fpsyg.2021.594255 (PMC8086429; doi:10.3389/fpsyg.2021.594255)

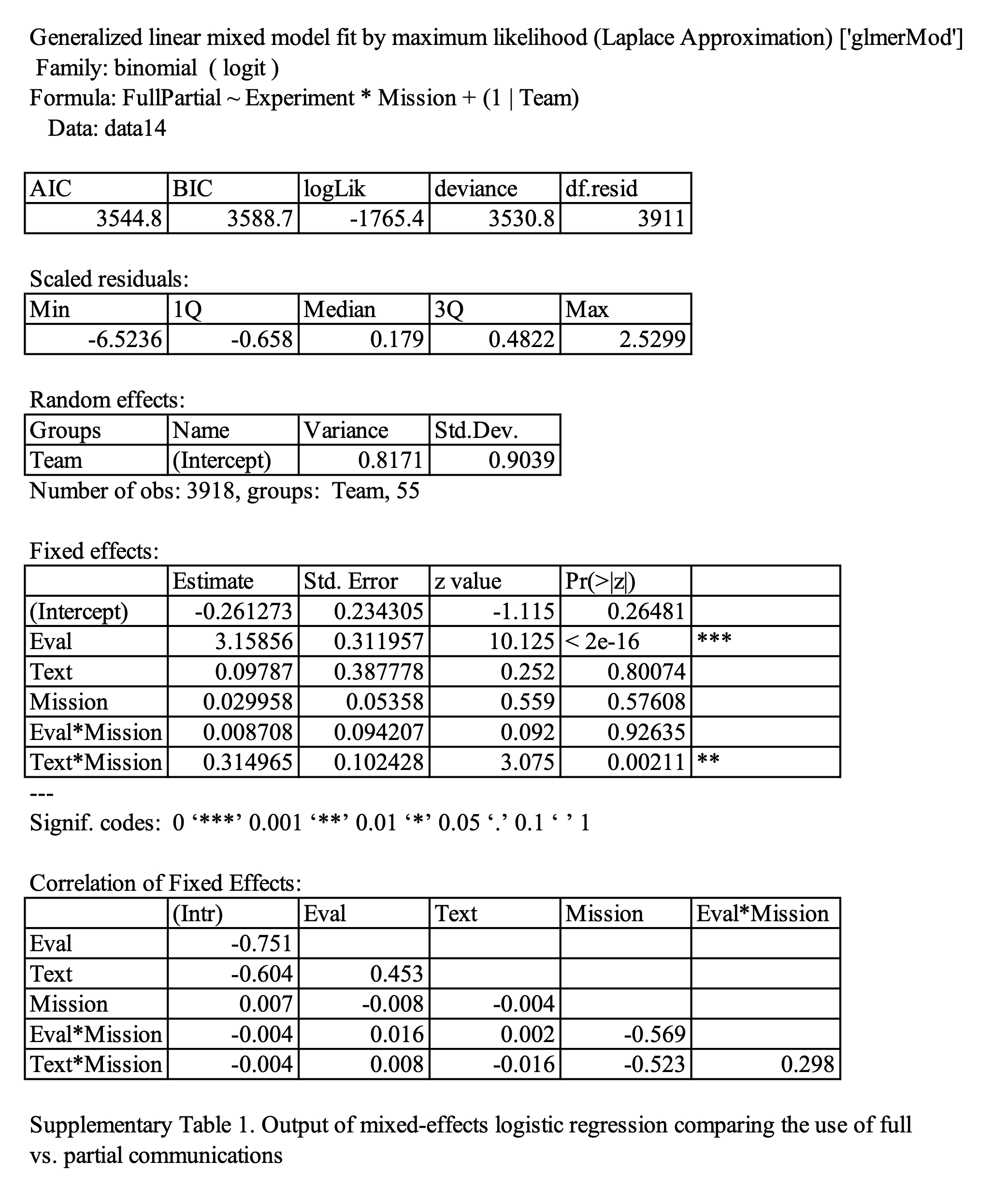

Supplement: Supplementary file 1 [file Image_1.jpg]

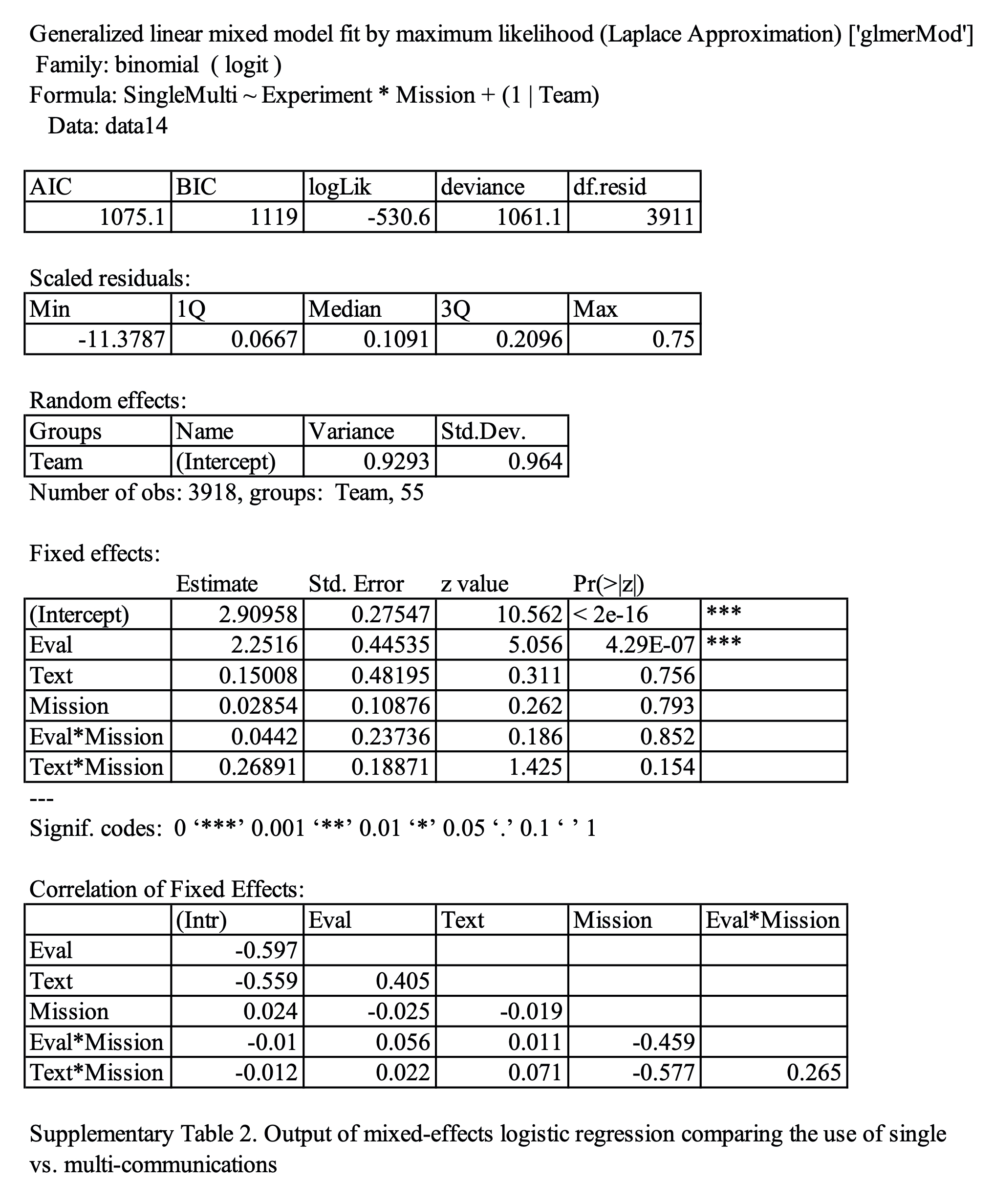

Supplement: Supplementary file 2 [file Image_2.jpg]

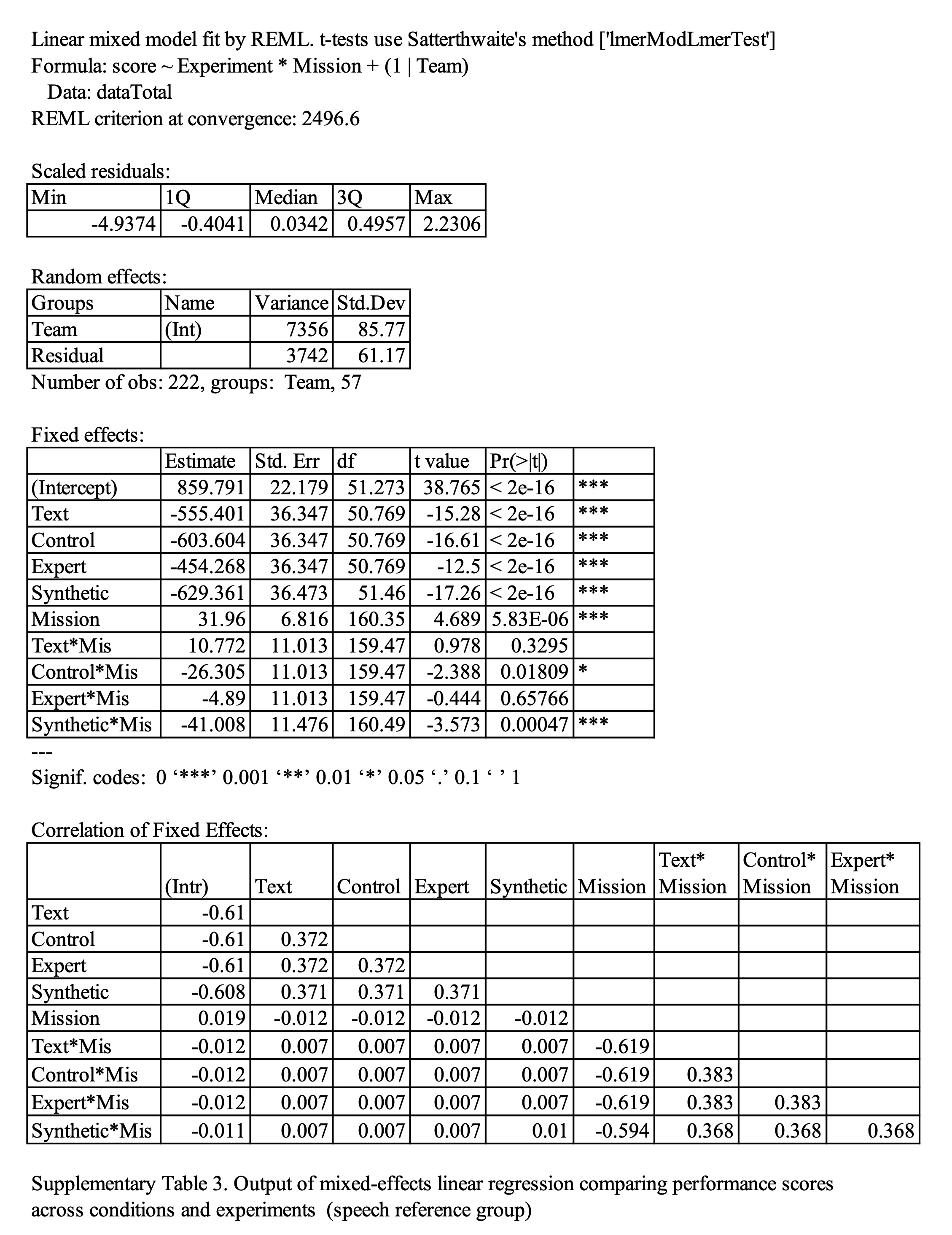

Supplement: Supplementary file 3 [file Image_3.jpg]

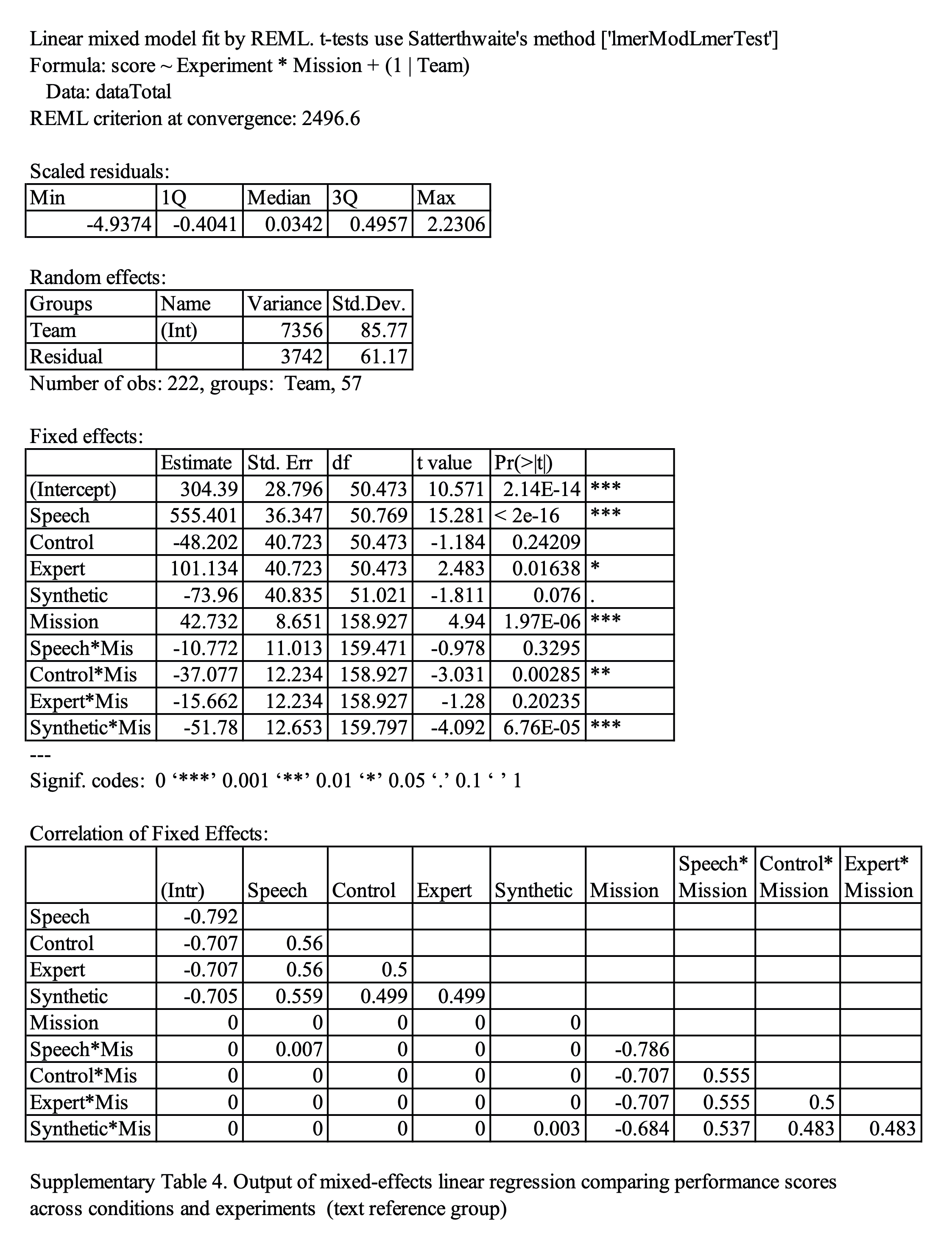

Supplement: Supplementary file 4 [file Image_4.jpg]
